# The general health of children and adolescents in Germany. Results of the cross-sectional KiGGS Wave 2 study and trends

**DOI:** 10.17886/RKI-GBE-2018-021

**Published:** 2018-03-15

**Authors:** Christina Poethko-Müller, Benjamin Kuntz, Thomas Lampert, Hannelore Neuhauser

**Affiliations:** Robert Koch Institute, Berlin, Department of Epidemiology and Health Monitoring

**Keywords:** GENERAL HEALTH, CHILDREN AND ADOLESCENTS, SUBJECTIVE HEALTH, HEALTH MONITORING

## Abstract

Data from KiGGS Wave 2 show that 95.7% of parents who participated in the survey rated the overall health of their 3 to 17 year-old children as very good or good. This proportion is higher than the figures identified by the KiGGS baseline study across all age groups. The proportion of children rated as in very good health is highest among 3 to 6 year-olds and decreases with age. Among 14 to 17 year-olds, the proportion of girls in very good health is well below the level found among boys. A pronounced social gradient is still clear from the data: the proportion of parents who assess the overall health of their children as very good or good rises with increasing social status. This highlights the need for strategies to reduce health inequalities that involve society as a whole, and the need for target group-specific measures in prevention and health promotion.

## Background

Good general health is an important resource in helping young people to successfully tackle the numerous developmental tasks they face during childhood and adolescence. Moreover, a good state of health – defined broadly – can also be understood as demonstrating that these developmental tasks have been tackled successfully. Adverse health effects that appear at an early age can continue from childhood through adolescence and into adulthood, and encourage the emergence of and reinforce long-term health problems [1].

Subjective assessments of general health are an integral aspect of many health surveys. Data collection is facilitated by means of a simple question. The comprehensive assessments of a person’s own or a child’s health are complex indicators as they include both objective and subjective aspects of health, and have no set time scale. However, studies of adults have repeatedly shown that self-rated health is a good predictor of morbidity in later life, increased uptake of health care and even mortality [2, 3]. There is also a correlation between self-rated health and physical and mental illnesses, psychological and social well-being, as well as health-related behaviour and the use of health services among children and adolescents [4, 5]. Nevertheless, studies that consider the prognostic value of general health as an indicator in children and adolescents are still rare. In Germany, data from the KiGGS cohort has shown that self-rated adolescent general health has a predictive value for the later onset of chronic illness and the use of health services, and that the information supplied by parents can also provide important indications about future health developments [6].

## Indicator and methodology

The German Health Interview and Examination Survey for Children and Adolescent (KiGGS) is part of the health monitoring programme undertaken at the Robert Koch Institute. The survey involves repeated cross-sectional surveys of children and adolescents aged between 0 and 17 (KiGGS cross-sectional study) that are representative of the German population. After carrying out the baseline study as an interview and examination survey (between 2003 and 2006) and KiGGS Wave 1 as an interview-based survey (between 2009 and 2012), KiGGS Wave 2 took place between 2014 and 2017 as a combined examination and interview survey.

**KiGGS Wave 2**

Second follow-up to the German Health Interview and Examination Survey for Children and Adolescents

**Data owner:** Robert Koch Institute

**Aim:** Providing reliable information on health status, health-related behaviour, living conditions, protective and risk factors, and health care among children, adolescents and young adults living in Germany, with the possibility of trend and longitudinal analyses

**Study design**: Combined cross-sectional and cohort study

**Cross-sectional study in KiGGS Wave 2**

**Age range:** 0-17 years

**Population:** Children and adolescents with permanent residence in Germany

**Sampling:** Samples from official residency registries - randomly selected children and adolescents from the 167 cities and municipalities covered by the KiGGS baseline study

**Sample size:** 15,023 participants

**KiGGS cohort study in KiGGS Wave 2**

**Age range:** 10-31 years

**Sampling:** Re-invitation of everyone who took part in the KiGGS baseline study and who was willing to participate in a follow-up

**Sample size:** 10,853 participants

**KiGGS survey waves**

► KiGGS baseline study (2003-2006), examination and interview survey
► KiGGS Wave 1 (2009-2012), interview survey
► KiGGS Wave 2 (2014-2017), examination and interview survey

More information is available at www.kiggs-studie.de/english

A detailed description of the methodology used in KiGGS Wave 2 can be found in New data for action. Data collection for KiGGS Wave 2 has been completed in issue S3/2017 as well as KiGGS Wave 2 cross-sectional study – participant acquisition, response rates and representativeness in issue 1/2018 of the Journal of Health Monitoring [9, 10].

KiGGS Wave 2 collected data via parental assessments of the general health of their 0 to 17 year-old children and through self-assessments of health made by 11 to 17 year-olds using a written questionnaire [7]. According to a wording recommended by the World Health Organization (WHO) [9], the participants were asked, ‘How is your child’s health in general?’ or, if applicable, ‘How is your health in general?’. The respondents were given a response scale consisting of five possible answers: ‘very good’, ‘good’, ‘fair, ‘bad’ and ‘very bad’.

This article employs information provided by parents on the general health of their 3 to 17 year-old children, as data is available for this entire age range, unlike the self-reported data collected from older children and adolescents. The analyses are based on valid data from 13,315 adolescents (6,682 girls, 6,633 boys). In the following, the five-step response scale is grouped into three categories (‘fair, ‘bad’ and ‘very bad’ were grouped into one category) and the data is arranged according to gender, age and socioeconomic status (SES) [10]. In addition, the response categories ‘very good’ and ‘good’ were summarised to aid comparison of data from the KiGGS baseline study with KiGGS Wave 2. The analyses were stratified by age and gender.

The calculations were carried out using a weighting factor that corrects deviations within the sample from the population structure with regard to age in years, gender, federal state, nationality and the parents’ educational distribution (Microcensus 2013 [11]).

A statistically significant difference between groups is assumed to have been demonstrated in cases where a p-value (after weighting and the survey design have been taken into account) was lower than 0.05.

## Results and discussion

The information provided by parents demonstrates that 95.7% of children and adolescents aged between 3 and 17 have good or very good health. If all age groups are considered together, no significant gender difference was found among the children with good or very good health. However, differences were identified according to age and gender in the five-step response categories, and this was particularly the case with very good health. Among both girls and boys, the proportion of children rated as having very good health is highest among 3 to 6 year-olds; this rate decreases with age (Table 1). However, parents of girls aged 10 or under are more likely to rate their general health as very good in comparison with boys.

As was the case with the data from 2003 to 2006 [12, 13], the proportion of girls aged between 14 and 17 with very good health (45.3%) is significantly lower than the rate identified among boys (52.4%). In this age group, the proportion of girls with very good health is lower than the proportion of those with good health; moreover, the proportion of girls aged 14 to 17 with fair or bad health is well above the level found among younger girls (Table 1). Similarly, a decline in the proportion of boys with very good health was also identified; however, as there was a particular increase in the proportion of boys with good health, the proportion of boys with fair or bad health across all age groups increased only slightly (Table 1).

The latest information provided by parents on the general health of adolescents supports the findings from the KiGGS baseline study, and, therefore, the self-assessments made by the adolescents themselves: girls aged between 14 and 17 rate their health as fair or very bad significantly more often than boys [14]. In addition, this difference in self-rated health between adolescent boys and girls is also clear from current data from KiGGS Wave 2 (data not shown). The data collected for the KiGGS study over the years demonstrate a phenomenon that has been identified in other countries: whereas a similar proportion of girls and boys aged 13 or below rate their health as fair or bad, about twice as many girls as boys do so during adolescence [1].

Debates about these issues focus on explanations such as the fact that developmental tasks not only become more complex during adolescence but also more gender-specific and that girls and boys face different forms of physiological and psychological stress during this time [15]. In addition, gendered differences in the perception and management of these demands and pressures are also under discussion as possible explanations [16, 17].

The data from KiGGS Wave 2, like the data from previous KiGGS surveys, emphasise that the chances of growing up in very good or good health are not evenly distribut ed: a highly pronounced social gradient still exists between boys and girls from families with high, medium and low social status. As a result, the proportion of parents who assess their children’s overall health as very good or good increases with rising social status [10].

**Figure 1:**
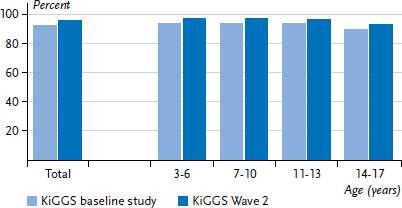
Prevalence of parent-rated very good or good general health according to age, comparing data from the KiGGS baseline study (n=7,173) and KiGGS Wave 2 (n=6,682) for 3 to 17 year-old girls Source: KiGGS baseline study (2003-2006), KiGGS Wave 2 (2014-2017)

**Figure 2:**
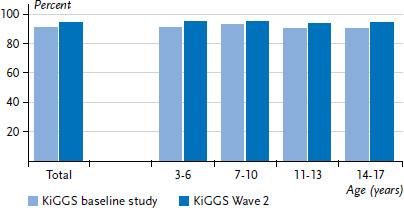
Prevalence of parent-rated very good or good general health according to age, comparing KiGGS baseline study (n=7,457) and KiGGS Wave 2 (n=6,633) for 3 to 17 year-old boys Source: KiGGS baseline study (2003-2006), KiGGS Wave 2 (2014-2017)

Over the last ten years, the data collected for the KiGGS study during its three survey periods – the KiGGS baseline study, KiGGS Wave 1 and KiGGS Wave 2 – show that parents are increasingly assessing the general health of the vast majority of children and adolescents in Germany in more favourable terms [13]. This is demonstrated by the fact that KiGGS Wave 2 (Figure 1 and Figure 2) identified a significantly higher rate than the KiGGS baseline study. However, an analysis that differentiates between the subjective and objective aspects of this trend cannot be conducted using data on self-rated health alone [18, 19]. Instead, overall developments in KiGGS indicators on physical and mental health, health-related quality of life and living conditions also need to be considered, as they provide valuable information for comprehensive health monitoring over time. Undoubtedly, however, subjective health remains an important integrative indicator that can be used to assess the health of children and adolescents in Germany – as the analyses differentiated according to gender, age and social status make clear. For example, current evidence suggests that parents with a low social status are significantly more likely to assess the health of their children as fair, bad, or very bad than parents from medium or high status groups. This underscores the need for strategies to reduce health inequalities that involve society as a whole, and the need for target group-specific measures in prevention and health promotion [10].

Overall, the population-based, cross-sectional data from KiGGS Wave 2 provide information about the current health of the population and factors associated with health and health-related behaviour. As such, they enable analyses of developments to be conducted over time. Research still needs to be undertaken into the factors that promote the healthy development of children and adolescents into young adulthood despite difficult individual social and health conditions. Also factors which could be used as starting points for target group-specific interventions need to be identified. Consequently, evaluations of the existing interview and examination data gathered recurrently from the participants of the KiGGS cohort are needed and additional data gained from a continued study of the KiGGS cohort will help to contribute towards resolving these issues in the future.

## Key statements

- More than 95% of parents who participated in KiGGS Wave 2 rate the general health of their 3 to 17 year-old children as ‘very good’ or ‘good’. This figure is higher in all age groups than the rate identified by the KiGGS baseline study.
- The proportion of children rated as in ‘very good’ health is highest among 3 to 6 year-olds and decreases with age.
- Parents of 14 to 17 year-old children rate the general health of girls as ‘very good’ much less frequently than they do with boys.
- The proportion of parents who rate their children’s overall health as ‘very good’ or ‘good’ increases with rising social status.

## Figures and Tables

**Table 1:** Prevalence of parent-rated general health according to gender, age and socioeconomic status (n=6,682 girls, n=6,633 boys) Source: KiGGS Wave 2 (2014-2017)

Very good			Good		Fair/Bad/Very bad	
	%	(95% CI)	%	(95% CI)	%	(95% CI)
**Girls (total)**	**58.0**	**(56.1-59.9)**	**37.9**	**(36.3-39.7)**	**4.0**	**(3.4-4.7)**
**Age**						
3-6 Years	67.0	(63.3-70.5)	30.2	(27.0-33.6)	2.7	(1.7-4.4)
7-10 Years	63.9	(60.7-67.0)	33.4	(30.4-36.5)	2.7	(1.9-3.7)
11-13 Years	57.0	(53.7-60.3)	39.5	(36.3-42.7)	3.5	(2.5-4.9)
14-17 Years	45.3	(42.2-48.3)	48.0	(44.8-51.2)	6.8	(5.3-8.5)
**Socioeconomic status**						
Low	50.4	(45.6-55.2)	42.8	(38.3-47.5)	6.8	(5.1-9.0)
Medium	56.7	(54.6-58.8)	39.4	(37.4-41.4)	3.9	(3.2-4.6)
High	71.3	(68.7-73.8)	27.6	(25.2-30.2)	1.0	(0.6-1.7)
**Boys (total)**	**56.2**	**(54.2-58.1)**	**39.2**	**(37.3-41.2)**	**4.6**	**(3.8-5.5)**
**Age**						
3-6 Years	61.9	(58.5-65.2)	34.0	(30.8-37.4)	4.1	(2.9-5.8)
7-10 Years	56.5	(53.2-59.8)	39.1	(35.9-42.4)	4.3	(3.0-6.4)
11-13 Years	54.0	(50.5-57.5)	40.8	(37.5-44.2)	5.2	(3.7-7.3)
14-17 Years	52.4	(48.8-56.0)	42.8	(39.3-46.5)	4.8	(3.3-6.9)
**Socioeconomic status**						
Low	46.7	(42.4-51.1)	44.8	(40.2-49.4)	8.5	(6.2-11.6)
Medium	56.0	(53.7-58.3)	39.7	(37.4-42.1)	4.2	(3.4-5.2)
High	66.1	(63.3-68.9)	32.2	(29.6-35.0)	1.6	(1.0-2.5)
**Total (Girls and Boys)**	**57.1**	**(55.6-58.5)**	**38.6**	**(37.3-40.0)**	**4.3**	**(3.8-4.9)**

CI=confidence interval
